# Identifying Patients with Poststroke Mild Cognitive Impairment by Pattern Recognition of Working Memory Load-Related ERP

**DOI:** 10.1155/2013/658501

**Published:** 2013-10-23

**Authors:** Xiaoou Li, Yuning Yan, Wenshi Wei

**Affiliations:** ^1^Shanghai Medical Instrumentation College, Shanghai 200093, China; ^2^School of Medical Instrument and Food Engineering, University of Shanghai for Science and Technology, Shanghai 200093, China; ^3^Department of Neurology, Huadong Hospital Affiliated to Fudan University, Shanghai 200040, China

## Abstract

The early detection of subjects with probable cognitive deficits is crucial for effective appliance of treatment strategies. This paper explored a methodology used to discriminate between evoked related potential signals of stroke patients and their matched control subjects in a visual working memory paradigm. The proposed algorithm, which combined independent component analysis and orthogonal empirical mode decomposition, was applied to extract independent sources. Four types of target stimulus features including P300 peak latency, P300 peak amplitude, root mean square, and theta frequency band power were chosen. Evolutionary multiple kernel support vector machine (EMK-SVM) based on genetic programming was investigated to classify stroke patients and healthy controls. Based on 5-fold cross-validation runs, EMK-SVM provided better classification performance compared with other state-of-the-art algorithms. Comparing stroke patients with healthy controls using the proposed algorithm, we achieved the maximum classification accuracies of 91.76% and 82.23% for 0-back and 1-back tasks, respectively. Overall, the experimental results showed that the proposed method was effective. The approach in this study may eventually lead to a reliable tool for identifying suitable brain impairment candidates and assessing cognitive function.

## 1. Introduction

Cognitive impairment after a stroke can affect the activities of daily living. Specifically, stroke patients are often associated with the working memory loss compared to age-matched healthy controls. The working memory is used for temporary storage and manipulation of the information and plays a key role in long-term memory, language, and execution function. Mild cognitive impairment (MCI) is common in poststroke patients [1], and it is widely considered to be the clinical transition stage between normal aging and dementia. Therefore, early MCI detection is of crucial importance for preventing poststroke dementia onset [2–6].

The accurate identification and assessment of cognitive function present a major clinical challenge. At present, the medical diagnosis of MCI is usually performed by some extensive tests including neuropsychological tests such as Mini-Mental State Examination (MMSE), neurological examination, and electrophysiological signal detection such as EEG [4, 7–9]. Due to its temporal resolution in the millisecond range as well as its noninvasiveness, wide availability, and relatively low costs, EEG is a popular measurement technique containing a lot of information about the human brain function and neurological disorders [10, 11]. It can also provide the objectivity and quantity evidence for the medical diagnosis [12, 13].

Prior EEG studies have concentrated on measuring scalp P300 event related potential (ERP) and EEG frequency power in cognitive impairment patients. The P300 component is typically elicited approximately 300 ms after each infrequent target stimulus, with reflecting the context updating and the categorization of relevant tasks [14–16]. Parameters extracted from ERP signals are of clinical interest because they are useful in differentiating the healthy controls from cognitive impairment patients [8, 11, 17]. In the time domain, the majority of studies on P300 in cognitive impairment have reported prolonged latencies and reduced amplitudes in visual or auditory modality [15, 18]. In the frequency domain, the spectral studies on the cognition have reported theta power changes due to increased demands on cognitive processes, such as the presentation of infrequent target stimulus in an oddball paradigm. Therefore, it is possible that the reduction of EEG theta power is a feature of cognitive impairment [2, 15, 19, 20].

Some studies have been motivated by the goal of using EEG to identify cognitive impairment patients with effective algorithms. Lehmann et al. explored the ability of a multitude of linear and nonlinear classification algorithms (i.e., linear discriminant analysis (LDA), neural network (NN), and support vector machine (SVM)) to discriminate between EEG signals of patients with varying degrees of cognitive impairment [9]. Dauwels et al. used LDA and quadratic discriminant analysis (QDA) to classify cognitive impairment [4]. Akrofi et al. studied the classification of cognitive impairment using Gaussian mixture model and selected features from relative average power and the coherence between intrahemispheric channel pairs [3]. Gallego-Jutglà et al. used theta band power and LDA to achieve the best accuracy for diagnosing cognitive impairment [21].

In those EEG-based classification algorithms, SVM based on structural risk minimization yields good performances in many applications, especially for solving problems with high dimension, nonlinearity, and small dataset. However, it is often unclear what the most suitable kernel in SVM is, and so the user may wish to combine several possible kernels. Multiple kernel learning SVM (MKL-SVM) is an efficient way of optimizing kernel weights [22, 23]. Compared with one single kernel SVM, MKL-SVM can enhance the interpretability of the decision function and improve the performance [24, 25].

Recently, nonlinear methods that include independent component analysis (ICA) and orthogonal empirical model decomposition (OEMD) have been proposed to extract parameters for the analysis and classification of EEG signals [11, 16]. ICA is a kind of blind source separation technique that extracts statistically independent sources called independent components (ICs) from a set of recorded signals [26]. OEMD is a self-adaptive signal processing and data driven method. Compared with classical time-frequency analysis methods, such as short time Fourier transform (STFT) and Wavelet decomposition, it is based on the local characteristic time scales of a signal and could decompose the signal into a set of complete orthogonal components called intrinsic mode functions (IMFs) which are determined by the signal itself without prior knowledge about the signal [26, 27]. OEMD can overcome the mode aliasing and avoid the occurrence of the fault mode [28].

In this study, we first applied the algorithm combining ICA and OEMD to the ERP data of stroke patients and healthy controls and used four types of features including P300 peak latency, P300 peak amplitude, root mean square (RMS), and theta frequency band power to separate stroke patients from healthy ones. Then the features and the evolutionary multiple kernel SVM (EMK-SVM) based on genetic programming (GP) were used to perform the recognition of stroke patients and healthy controls based on working memory tasks. These tasks that may elicit a P300 ERP component were 0-back and 1-back tasks. 

## 2. Materials and Methods

**Figure 1:**
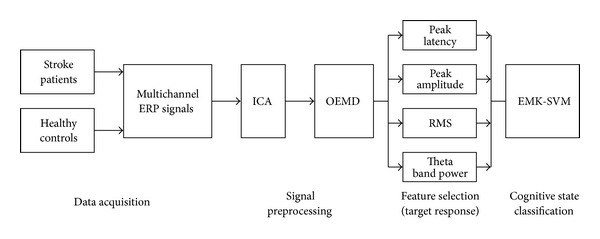
The general diagram of the proposed methodology.

We proposed a classification approach of stroke patients and healthy controls, as illustrated in Figure 1. The presented approach consisted of three main parts. (1) The preprocessing algorithm combining ICA and OEMD was used to extract independent source components from the 18-channel ERP signals. (2) Four types of features including P300 peak latency, P300 peak amplitude, RMS, and theta frequency band power were estimated, and they were differently chosen to compose a feature vector for further classification. (3) EMK-SVM was employed to perform the working memory task classification, and the classification accuracies were used to evaluate the performance of the proposed algorithm.

### 2.1. ERP Recordings

Consecutive patients aged 50 years or older with a first-ever acute ischemic stroke at Huadong Hospital Affiliated to Fudan University between May 2012 and January 2013 were recruited. All patients underwent neuropsychological and neuroimaging assessments, and those who met the criteria for vascular MCI were included (*n* = 13) [29, 30]. 13 age- and sex-matched healthy controls were enrolled in this cross-sectional study. All subjects were right handed and had normal vision. This study was approved by Huadong Hospital Affiliated to Fudan University Ethics Board, and all subjects gave written, informed consents before participation.

**Figure 2:**
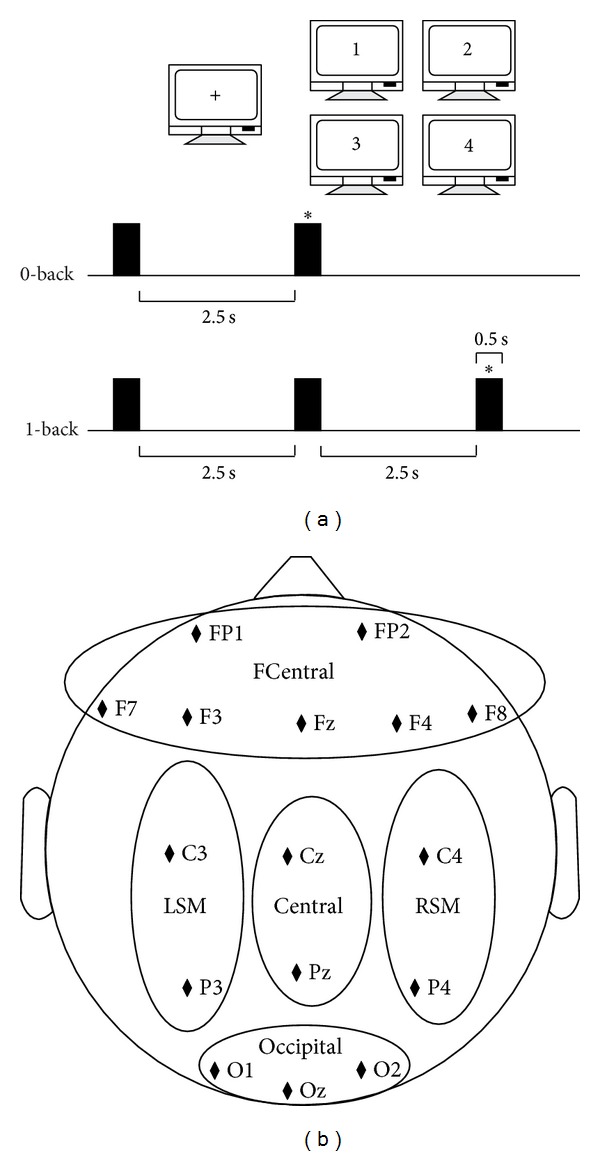
*N*-back task timeline and electrode positions. Here trial time sequences for 0-back and 1-back conditions. Black squares represent each stimulus in the task. The symbol ∗ stands for the target number during each trial. Five brain regions: frontocentral (FCentral): FP1, FP2, F7, F3, Fz, F4, and F8; left sensorimotor (LSM): C3 and P3; central: Cz and Pz; right sensorimotor (RSM): C4 and P4; and occipital: O1 and O2.

**Figure 3:**
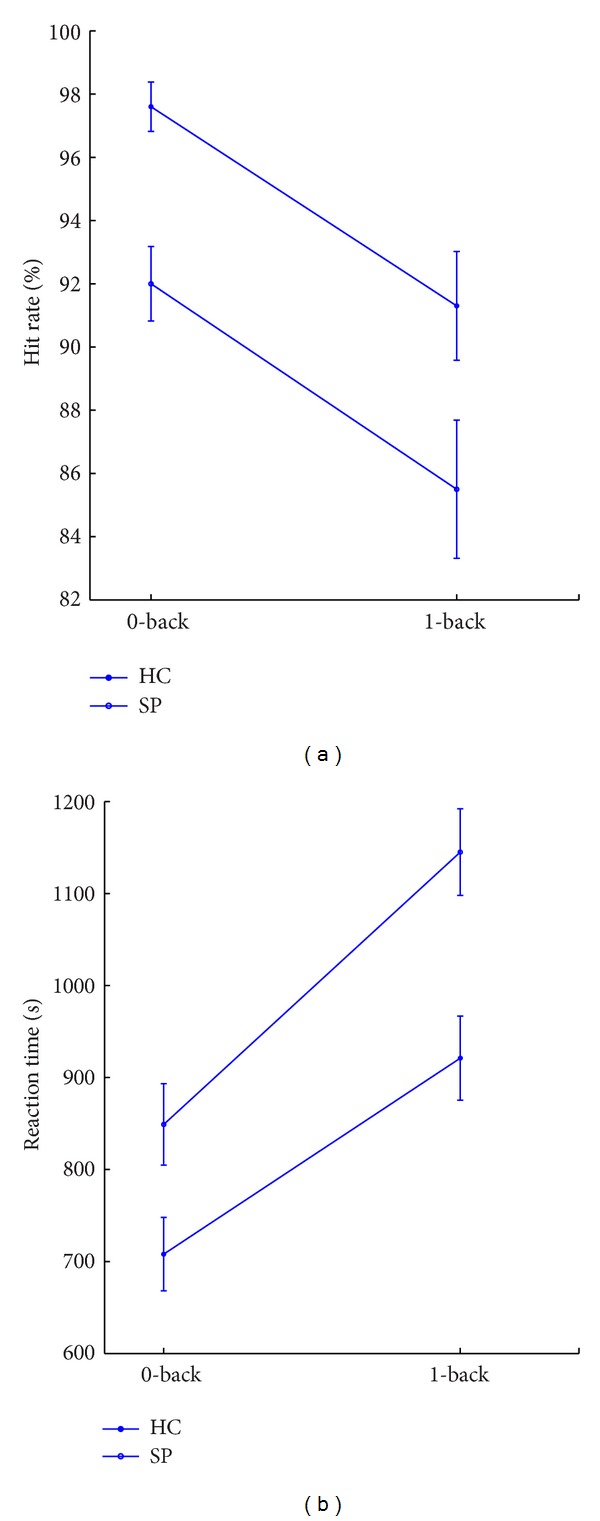
Task behavior performances with hit rate and reaction time. Here HC represents healthy control and SP represents stroke patient.

As shown in Figure 2, the working memory was assessed using a verbal *N*-back task [5, 31]. A pseudorandom set of 4 digit numbers was displayed on a monitor, and the subjects were instructed to determine whether specific digit one appeared on the screen (0-back task); or the currently displayed number at any given time had been already displayed in the previous presentation (1-back task). Stimuli consisted in a 0.5 sec. Inter-stimulus interval (ISI) was 2.5 sec in all conditions. Subjects had to distinguish between targets and nontargets by pressing a keyboard. Continuous ERP signals were acquired using an EEG/ERP amplifier system (NATION Inc.). For all ERP recordings, 18 electrodes were placed according to the 10–20 international system. The chosen electrode positions were EOG1, EOG2, Fp1, Fp2, F3, F4, F7, F8, Fz, C3, Cz, C4, P3, Pz, P4, O1, Oz, and O2 (see Figure 2). The data were sampled at 256 Hz. Signals were recorded for 120 s during each task. Each task was repeated for three sessions. Each session contained 40 trials with a 1 : 1 target/nontarget relation. Namely, the total number of targets was 60, the same as that of nontargets. The hit rate and the reaction time were measured, as shown in Figure 3.

### 2.2. Preprocessing Algorithms

The difficulty in developing a classification system based on EEG is to discriminate the responses from the background noise reliably because the signals are relatively weak and interfered easily by the artifacts, such as electromagnetic interference, powerline noise, EOG, EMG, ECG, and subject movements. Some preprocessing algorithms have been applied to the EEG data in order to extract more informative features, which can be used as inputs to a classifier to improve the classification accuracy of task-related activity, such as ICA and OEMD [32, 33]. The EMD method may encounter the difficulty of mode mixing, and it is mainly caused by noise, boundary effect, and so forth. This type of mixing will lead to the presence of several components of the signal of interest on the same IMF, which can cause the difficulty in the physical discriminant of each mode. To solve these problems, ICA and OEMD were combined in this study. we first removed the artifacts from the given ERP signals to extract statistically independent sources by independent component analysis (ICA) and then decomposed them to extract real IMF components using OEMD algorithm.

#### 2.2.1. Independent Component Analysis

ICA was applied on the entire collection of raw ERP signals *y*(*t*) = [*y*_1_(*t*),…,*y*_*L*_(*t*)]^*T*^, where *L* indicated the number of channels on the scalp. The goal of ICA is to find an unmixing matrix *W* that initially produces the ERP signals *y*(*t*) based on statistically independent sources *s*(*t*) in the matrix form *u* = *Wy* → *s*. In contrast to correlation-based transformations such as principal component analysis (PCA), ICA reduces the statistical dependencies of the signals and attempts to make them as independent as possible. This technique has shown great promise for analyzing EEG recordings [16, 34–36]. There are many ways for learning *W*. We used the extended Infomax algorithm which minimizes the mutual information among the data projections in order to achieve the independence.

The learning rule of the unmixing matrix *W* is [36]

(1) $$\begin{array}{c} ∆W\propto \left[I-K\tan h\left(u\right)u^{T}-uu^{T}\right]W,\\ W\left(m+1\right)=W\left(m\right)+\mu ∆W\left(m\right),\\ k_{i}=1\text{:}\;\;\text{super-Gaussian},\\ k_{i}=-1\text{:}\;\;\text{sub-Gaussian}, \end{array}$$

where *k*_*i*_ are elements of the *N*-dimensional diagonal matrix *K*, *m* is the iteration number, and *μ* is the step size. The switching parameter *k*_*i*_ can be derived from the variation of the kurtosis sign. *k*_*i*_ can be obtained as

(2) ki=sign⁡(E(ui4)−3(E(ui2))2(E(ui2))2)=sign⁡(E(ui4)(E(ui2))2−3).

#### 2.2.2. Orthogonal Empirical Model Decomposition

EMD is a data-adapted interactive method, which can decompose any complicated time series into additive components with multiscale features; that is,

(3) $$\begin{array}{c} f\left(t\right)=\overset{M}{\underset{j=1}{\sum }}g_{oj}\left(t\right)+r_{M+1}\left(t\right). \end{array}$$

These components are denoted by IMFs, where *M* is the number of IMFs, *g*_*oj*_(*t*) is the *m*th IMF, and *r*_*M*+1_(*t*) is the final residue [37].

One of the major drawbacks of the original EMD algorithm is that the IMFs are not strictly orthogonal to each other [27], which can cause the energy leakage while decomposing. Therefore, it is necessary to perform the orthogonal processing for the IMFs from EMD in order to obtain the completely orthogonal IMFs [38].

In particular, once the first IMF is derived, define *g*_1_(*t*) = *g*_*o*1_(*t*), which is the smallest temporal scale in *f*(*t*). To determine the rest of the IMFs, generate the residue *r*_1_(*t*) = *f*(*t*) − *g*_1_(*t*). *r*_1_(*t*) can be treated as the new signal and the EMD decomposing is performed for the second IMF *g*_*o*2_(*t*). In order to achieve the orthogonal component, *g*_1_(*t*) has to be subtracted from *g*_*o*2_(*t*); that is,

(4) $$\begin{array}{c} g_{2}\left(t\right)=g_{o2}\left(t\right)-\beta_{21}g_{1}\left(t\right), \end{array}$$

where *g*_2_(*t*) is the second orthogonal IMF and *β*_21_ is the orthogonal parameter between *g*_*o*2_(*t*) and *g*_1_(*t*). With the orthogonality between *g*_2_(*t*) and *g*_1_(*t*), *β*_21_ can be obtained as

(5) $$\begin{array}{c} \overset{T}{\underset{0}{\int }}g_{1}\left(t\right)g_{2}\left(t\right)=\overset{T}{\underset{0}{\int }}g_{1}\left(t\right)g_{o2}\left(t\right)-\beta_{21}\overset{T}{\underset{0}{\int }}g_{1}^{2}\left(t\right)=0,\\ \beta_{21}=\frac{\int_{0}^{T}g_{1}\left(t\right)g_{o2}\left(t\right)}{\int_{0}^{T}g_{1}^{2}\left(t\right)}. \end{array}$$

The above process is repeated until the expected index number of IMF is met [11, 38].

#### 2.2.3. Ensemble ICA-OEMD

First, ICA based on the extended Infomax algorithm was applied to ERP signals, and the independent components were extracted. Second, OEMD was performed for each obtained source, and a set of orthogonal IMFs was derived, in which only the IMF of interest based on theta frequency band power and the peak between 200 ms and 450 ms was selected. The combining decomposition is adaptive to the time and frequency content of the data themselves and can separate original ERP signals into orthogonal components with different time scales. Meanwhile, the orthogonality property implies that different IMFs do not have similar frequency content [26]. The algorithm is described in Algorithm 1.

### 2.3. Feature Extraction

The efficient feature extraction from multichannel working memory task EEG signals is a major component for the cognitive state classification. In this study, four types of features including P300 peak latency, P300 peak amplitude, RMS, and theta frequency band power were chosen. They are classical, quantitative ERP measures that are commonly used in this field [14]. In particular, we extracted theta frequency band data of working memory task EEG signals through the wavelet packet transform (WPT) decomposition (4 levels) with the wavelet basis db4 and then calculated the energy spectrum.

### 2.4. Classification Algorithm

SVM is a powerful approach for pattern recognition especially for high dimensional, nonlinear problems. Recent developments on SVM have shown that it is necessary to consider multiple kernels [22]. This provides flexibility and reflects the fact that typical learning problems often involve multiple, heterogeneous data sources. Although the MKL-SVM algorithm is shown to improve the classification performance effectively, it relies more on the empirical kernel functions (e.g., polynomial function and radial basis function) and parameters (e.g., degree and Gaussian width), which can affect its effectiveness because different functions and parameters may result in different performances. A potential solution is to use GP to evolve the kernels and associated parameters automatically [39, 40].

GP is an evolutionary algorithm inspired by biological evolution, where each individual is a computer program represented as a tree structure. These computer programs that solve a given problem are genetically bred. This breeding is done by using the genetic operations from Darwinian principle, such as selection, crossover, and mutation. The evolutionary process is repeated over many generations until the fittest individual computer program is found [40].

In this study, we evolved more effective kernel function using GP and kernel closure properties, where each tree structure represented a multiple kernel function [41]. EMK-SVM is described in Algorithm 2.

The algorithm was based on GP-kernel and SVM. First, GP started with an initial population of randomly generated computer programs which are composed of functions and terminals, and their individuals were some nonlinear combination trees of kernel functions. Second, the SVM learning was performed for each individual tree. The following steps were performed iteratively until the termination criterion has been satisfied.

- Execute each program in the population and assign it a fitness value (classification accuracy) according to how well it solves the classification with SVM.
- Create a new population by applying the genetic operations.

Third, the best kernel function that appeared in any generation was designated as the result.

Some settings of control parameters used in this study are given in Table 1.

## 3. Results and Discussion

### 3.1. General Classification Performance

In this study, 16-channel ERP signals were classified via a 5-fold cross-validation. First, the ERP data were partitioned into 5 equally sized folds. Second, 5 iterations of training and validation were performed such that within each iteration a different fold of the ERP data was held out for validation while the remaining 4 folds were used for learning. Finally, the classification results from 5 folds were averaged to produce the classification accuracy. Four types of features, including P300 peak latency, P300 peak amplitude, RMS, and theta frequency band power, were extracted for the classification using the EMK-SVM algorithm.

The results of the *F*-test and *t*-test are shown in Table 2. For 0-back and 1-back tasks, the differences between stroke patients and healthy controls were significant. There were differences of P300 latency, P300 amplitude, RMS, and theta band power in the stroke patients compared with healthy ones. In particular, the differences of P300 amplitude and RMS between stroke patients and healthy controls in 1-back task were larger. 

For 0-back and 1-back tasks, the multiple comparison tests were also performed to determine which were significantly different from other groups on the basis of Table 2. The results of the multiple comparison tests with the Bonferroni procedure are shown in the Table 3. For four types of features, the differences between stroke patient group and healthy control group were obvious, which were consistent with the results in Table 2. These significant differences provide a basis for the further classification.

Table 4 shows the results achieved with four selected features using the EMK-SVM classifier distinguishing stroke patients versus healthy controls. The classification results obtained in 0-back and 1-back tasks ranged from 75% to 91.67%. In 0-back task, the accuracy for RMS or theta band power was the highest, 91.67%, while that for peak latency was the lowest, 78.4%. In 1-back task, the accuracy for theta band power was the highest, 82.23%, while that for peak latency was the lowest, 75%. As mentioned above, the general classification accuracy of 0-back task was higher than that of 1-back task. It can be seen that theta band power is the best feature used for the classification.

### 3.2. Classifier Results

**Figure 4:**
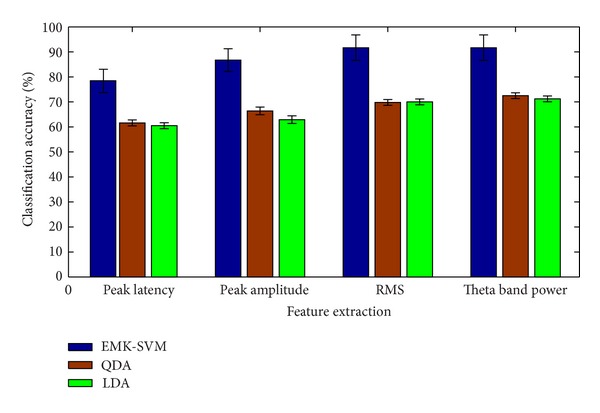
Accuracy comparisons of 0-back task classification.

**Figure 5:**
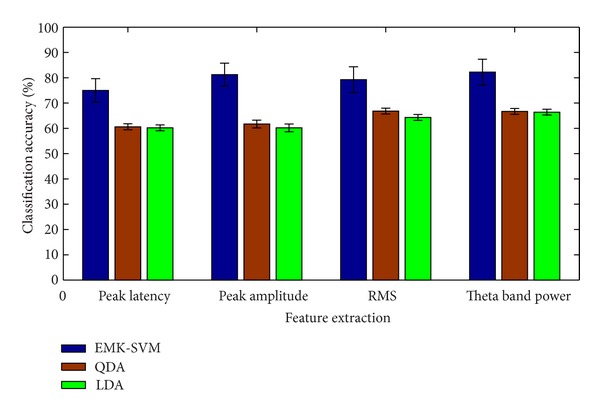
Accuracy comparisons of 1-back task classification.

Figures 4 and 5 show the classification comparison results of 0-back and 1-back tasks between the proposed algorithm and another two state-of-the-art algorithms (QDA and LDA) with the same features. As has been shown, the classification results based on EMK-SVM were better than those based on QDA and LDA.

EMK-SVM based on the parameters listed in Table 1 is performed for automatic classification of stroke patients and healthy controls. An initial population of 100 kernel function trees was created and then iteratively proceeded through 100 generations with the genetic operations. The initial generation consisted of highly unfit individuals. The intermediate generations contained a few somewhat fit individuals. The final generation of each run contained at least one individual that was effective in solving the classification problem. The optimal kernel functions are shown in Table 5. For example, the optimal kernel function for 0-back task classification with theta band power feature was exp⁡⁡(−1.68 *K*_Poly_^2^ *K*_RBF_^2^), which occured in generation 79. 

## 4. Conclusions

In this paper, we presented the EMK-SVM algorithm for ERP-based signal classification for stroke patients and healthy controls with four features (i.e., P300 peak latency, P300 peak amplitude, RMS, and theta frequency band power). The proposed method had better performance than other typical methods (i.e., QDA and LDA). It achieved above 78.4% accuracy for 0-back task and above 75% for 1-back task. The statistical test results showed that the differences of selected features were significant. Therefore, it provides a powerful tool to assess cognitive function. 

In sum, it is an effective method to implement working memory task-based BCI based on cognitive impairment. We applied the preprocessing algorithm combining ICA and OEMD to extract more informative features and also applied the recognition algorithm combining SVM and GP to discover better kernels to improve the classification accuracy. This study provides theoretical and experimental basis of the quantity diagnosis for cognitive impairment. It is helpful for the intelligent identification of cognitive function and appropriate rehabilitation training.

## Figures and Tables

**Algorithm 1:**
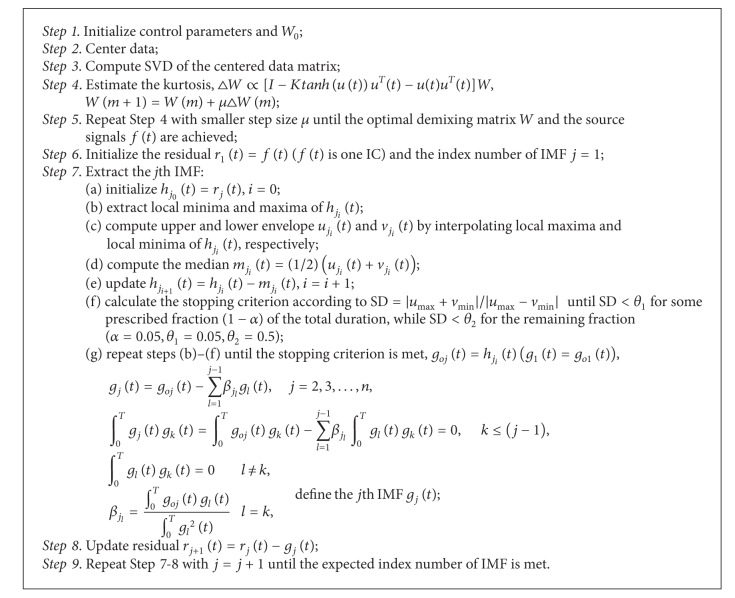
ICA-OEMD.

**Algorithm 2:**
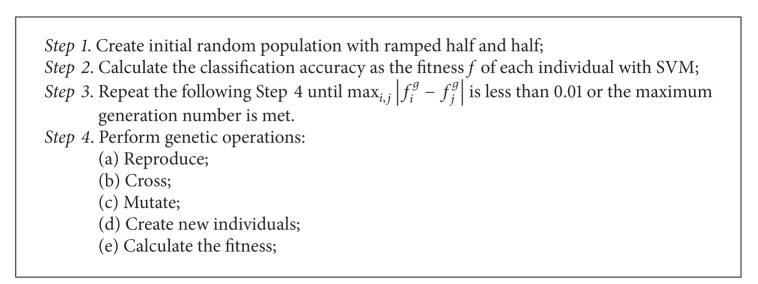
EMK-SVM.

**Table 1:** Parameters for evolutionary multiple kernel learning.

Parameter	Setting
Function set	FS = {+, ×, exp⁡}
Terminal set	TS = {*K*_Poly_, *K*_RBF_, α}
	*K*_Pol_ = *xy* + 1
	*K*_RBF_ = exp⁡(−||*x*−*y*||^2^/2)
	α ∈ [−1,1]
Population size	pop_size = 100
Maximum generation	max_gen = 100
Maximum depth	max_depth = 8
Reproduction probability	*P*_*r*_ = 0.1
Crossover probability	*P*_*c*_ = 0.85
Mutation probability	*P*_*m*_ = 0.1

**Table 2:** Statistical test results for 0-back and 1-back tasks with different features.

Classification tasks	Features	*F*-test		*t*-test	
		*F*	*P*	*t*	*P*
0-back (SP-HC)	Peak latency	4.91	<0.05	2.22	<0.05
	Peak amplitude	4.88	<0.05	2.21	<0.05
	RMS	4.00	<0.05	2.43	<0.05
	Theta band power	3.65	<0.05	1.91	<0.05

1-back (SP-HC)	Peak latency	3.53	<0.05	1.88	<0.05
	Peak amplitude	9.00	<0.01	2.99	<0.01
	RMS	7.56	<0.01	2.75	<0.01
	Theta band power	4.16	<0.05	2.53	<0.05

**Table 3:** Statistical test results for 0-back and 1-back tasks with the multiple comparison.

Four comparison groups	Features	*F*-test		Significant difference between groups
		*F*	*P*	
0-back (SP), 0-back (HC), 1-back (SP), 1-back (HC)	Peak latency	5.21	<0.01	0-back (SP) and 0-back (HC), 1-back (SP) and 1-back (HC)
0-back (SP), 0-back (HC), 1-back (SP), 1-back (HC)	Peak amplitude	2.86	<0.05	0-back (SP) and 0-back (HC), 1-back (SP) and 1-back (HC)
0-back (SP), 0-back (HC), 1-back (SP), 1-back (HC)	RMS	8.14	<0.01	0-back (SP) and 0-back (HC), 1-back (SP) and 1-back (HC), 0-back (SP) and 1-back (SP)
0-back (SP), 0-back (HC), 1-back (SP), 1-back (HC)	Theta band power	4.25	<0.01	0-back (SP) and 0-back (HC), 1-back (SP) and 1-back (HC), 0-back (SP) and 1-back (SP)

**Table 4:** Classification accuracies for 0-back and 1-back tasks with different features (%).

Classification tasks	Features	Classification accuracies (%)
0-back (SP-HC)	Peak latency	78.40
	Peak amplitude	86.70
	RMS	91.67
	Theta band power	91.67

1-back (SP-HC)	Peak latency	75.00
	Peak amplitude	81.25
	RMS	79.25
	Theta band power	82.23

**Table 5:** The optimal kernel functions for the classification tasks.

Classification tasks	Features	Optimal kernel functions	Generation number
0-back (SP-HC)	Peak latency	*K*_Poly_ + *K*_RBF_ − 1.736	73
	Peak amplitude	*K*_Poly_ + exp⁡(⁡−0.351*K*_RBF_) + exp⁡(*K*_RBF_^2^)	62
	RMS	exp⁡⁡(0.866)(*K*_Poly_ + *K*_RBF_) + *K*_Poly_^2^ *K*_RBF_^2^	69
	Theta band power	exp⁡(−1.68*K*_Poly_^2^ *K*_RBF_^2^)	79

1-back (SP-HC)	Peak latency	exp⁡(−0.628*K*_RBF_exp⁡⁡(*K*_RBF_))	59
	Peak amplitude	exp⁡(−0.923*K*_RBF_(*K*_Poly_ + *K*_RBF_)exp⁡⁡(*K*_RBF_))	79
	RMS	exp⁡(−0.598*K*_RBF_(*K*_Poly _ + *K*_RBF_))	81
	Theta band power	0.74*K*_Poly_(*K*_Poly_ + *K*_RBF_)exp⁡(−0.446 + *K*_RBF_)	62

## References

[1] Sachdev PS, Brodaty H, Valenzuela MJ (2006). Clinical determinants of dementia and mild cognitive impairment following ischaemic stroke: the sydney stroke study. *Dementia and Geriatric Cognitive Disorders*.

[2] Liu ZY, Bai LJ, Dai RW Exploring the effective connectivity of resting state networks in mild cognitive impairment: an fMRI study combining ICA and multivariate Granger causality analysis.

[3] Akrofi K, Pal R, Baker MC, Nutter BS, Schiffer RW Classification of Alzheimer’s disease and mild cognitive impairment by pattern recognition of EEG power and coherence.

[4] Dauwels J, Vialatte F, Latchoumane C, Jeong J, Cichocki A EEG synchrony analysis for early diagnosis of Alzheimer’s disease: a study with several synchrony measures and EEG data sets.

[5] Jo JM, Kim Y-H, Ko M-H, Ohn SH, Joen B, Lee KH (2009). Enhancing the working memory of stroke patients using tDCS. *American Journal of Physical Medicine and Rehabilitation*.

[6] Baker MC, Akrofi K, Schiffer R (2008). EEG patterns in mild cognitive impairment (MCI) patients. *The Open Neuroimaging Journal*.

[7] Lee DR, Taylor J-P, Thomas AJ (2012). Assessment of cognitive fluctuation in dementia: a systematic review of the literature. *International Journal of Geriatric Psychiatry*.

[8] Tosun D, Mojabi P, Weiner MW, Schuff N (2010). Joint analysis of structural and perfusion MRI for cognitive assessment and classification of Alzheimer’s disease and normal aging. *NeuroImage*.

[9] Lehmann C, Koenig T, Jelic V (2007). Application and comparison of classification algorithms for recognition of Alzheimer’s disease in electrical brain activity (EEG). *Journal of Neuroscience Methods*.

[10] Haufe S, Nikulin VV, Müllera KRM (2013). critical assessment of connectivity measures for EEG data: a simulation study. *NeuroImage*.

[11] Bajaj V, Pachori RB (2012). Classification of seizure and nonseizure EEG signals using empirical mode decomposition. *IEEE Transactions on Information Technology in Biomedicine*.

[12] Dauwels J, Vialatte F, Cichocki A (2010). Diagnosis of Alzheimer’s disease from EEG signals: where are we standing?. *Current Alzheimer Research*.

[13] Jeong J (2004). EEG dynamics in patients with Alzheimer’s disease. *Clinical Neurophysiology*.

[14] Mak JN, McFarland DJ, Vaughan TM (2012). EEG correlates of P300-based brain-computer interface (BCI) performance in people with amyotrophic lateral sclerosis. *Journal of Neural Engineering*.

[15] Kiiski H, Reilly RB, Lonergan R (2012). Only low frequency event-related EEG activity is compromised in multiple sclerosis: insights from an independent component clustering analysis. *Open Access Available Online*.

[16] Zervakis M, Michalopoulos K, Iordanidou V, Sakkalis V (2011). Intertrial coherence and causal interaction among independent EEG components. *Journal of Neuroscience Methods*.

[17] Wang YP, Zhang XL, Huang JJ (2013). Associations between EEG beta power abnormality and diagnosis in cognitive impairment post cerebral infarcts. *Journal of Molecular Neuroscience*.

[18] Dubovik S, Bouzerda-Wahlen A, Nahum L (2013). Adaptive reorganization of cortical networks in Alzheimer's disease. *Clinical Neurophysiology*.

[19] Dauwels J, Vialatte F, Musha T, Cichocki A (2010). A comparative study of synchrony measures for the early diagnosis of Alzheimer’s disease based on EEG. *NeuroImage*.

[20] Besthorn C, Zerfass R, Geiger-Kabisch C (1997). Discrimination of Alzheimer’s disease and normal aging by EEG data. *Electroencephalography and Clinical Neurophysiology*.

[21] Gallego-Jutglà E, Elgendi M, Vialatte F Diagnosis of Alzheimer's disease from EEG by means of synchrony measures in optimized frequency bands.

[22] Chen X, Wang ZJ (2013). Pattern recognition of number gestures based on a wireless surface EMG system. *Biomedical Signal Processing and Control*.

[23] Gönen M, Alpaydin E (2011). Multiple kernel learning algorithms. *Journal of Machine Learning Research*.

[24] Sonnenburg S, Rätsch G, Schäfer C, Schölkopf B (2006). Large scale multiple kernel learning. *Journal of Machine Learning Research*.

[25] Lanckriet GRG, Cristianini N, Bartlett P, El Ghaoui L, Jordan MI (2004). Learning the kernel matrix with semidefinite programming. *Journal of Machine Learning Research*.

[26] Mijović B, De Vos M, Gligorijević I, Taelman J, Van Huffel S (2010). Source separation from single-channel recordings by combining empirical-mode decomposition and independent component analysis. *IEEE Transactions on Biomedical Engineering*.

[27] Lei YG, Lin J, He ZJ (2013). A review on empirical mode decomposition in fault diagnosis of rotating machinery. *Mechanical Systems and Signal Processing*.

[28] Ren QS, Yi Q, Fang MY Fast implementation of orthogonal empirical mode decomposition and its application into singular signal detection.

[29] Petersen RC, Smith GE, Waring SC, Ivnik RJ, Tangalos EG, Kokmen E (1999). Mild cognitive impairment: clinical characterization and outcome. *Archives of Neurology*.

[30] Sachdev PS, Chen X, Brodaty H, Thompson C, Altendorf A, Wen W (2009). The determinants and longitudinal course of post-stroke mild cognitive impairment. *Journal of the International Neuropsychological Society*.

[31] Missonnier P, Deiber M-P, Gold G (2007). Working memory load-related electroencephalographic parameters can differentiate progressive from stable mild cognitive impairment. *Neuroscience*.

[32] Sweeney-Reed CM, Nasuto SJ (2007). A novel approach to the detection of synchronisation in EEG based on empirical mode decomposition. *Journal of Computational Neuroscience*.

[33] Chiappa S, Barber D (2006). EEG classification using generative independent component analysis. *Neurocomputing*.

[34] Akhtar MT, Jung TP, Makeig S Recursive independent component analysis for online blind source separation.

[35] Brunner C, Naeem M, Leeb R, Graimann B, Pfurtscheller G (2007). Spatial filtering and selection of optimized components in four class motor imagery EEG data using independent components analysis. *Pattern Recognition Letters*.

[36] Lee T-W, Girolami M, Sejnowski TJ (1999). Independent component analysis using an extended infomax algorithm for mixed subgaussian and supergaussian sources. *Neural Computation*.

[37] Huang B, Kunoth A (2013). An optimization based empirical mode decomposition scheme. *Journal of Computational and Applied Mathematics*.

[38] Lou M, Huang T (2007). Orthogonal empirical mode decomposition. *Journal of Tongji University*.

[39] Sullivan K, Luke S Evolving kernels for support vector machine classification.

[40] Koza JR (1994). Genetic programming as a means for programming computers by natural selection. *Statistics and Computing*.

[41] Espejo PG, Ventura S, Herrera F (2010). A survey on the application of genetic programming to classification. *IEEE Transactions on Systems, Man and Cybernetics*.

